# Antibiotic Resistance Genes in Phage Particles from Antarctic and Mediterranean Seawater Ecosystems

**DOI:** 10.3390/microorganisms8091293

**Published:** 2020-08-24

**Authors:** Pedro Blanco-Picazo, Gabriel Roscales, Daniel Toribio-Avedillo, Clara Gómez-Gómez, Conxita Avila, Elisenda Ballesté, Maite Muniesa, Lorena Rodríguez-Rubio

**Affiliations:** 1 Department of Genetics, Microbiology and Statistics, University of Barcelona, Diagonal 643, Annex, Floor 0, 08028 Barcelona, Spain; p.blanco@ub.edu (P.B.-P.); gascheb@gmail.com (G.R.); d.toribio.avedillo@ub.edu (D.T.-A.); claragg1995@gmail.com (C.G.-G.); eballeste@ub.edu (E.B.); 2 Department of Evolutionary Biology, Ecology and Environmental Sciences (BEECA), Faculty of Biology, and Biodiversity Research Institute (IrBIO), University of Barcelona, Diagonal 643, 08028 Barcelona, Spain; conxita.avila@ub.edu

**Keywords:** bacteriophages, shellfish, fish, transduction, ARG, horizontal gene transfer

## Abstract

Anthropogenic activities are a key factor in the development of antibiotic resistance in bacteria, a growing problem worldwide. Nevertheless, antibiotics and resistances were being generated by bacterial communities long before their discovery by humankind, and might occur in areas without human influence. Bacteriophages are known to play a relevant role in the dissemination of antibiotic resistance genes (ARGs) in aquatic environments. In this study, five ARGs (*bla*_TEM_, *bla*_CTX-M-1_, *bla*_CTX-M-9_, *sul1* and *tetW*) were monitored in phage particles isolated from seawater of two different locations: (i) the Mediterranean coast, subjected to high anthropogenic pressure, and (ii) the Antarctic coast, where the anthropogenic impact is low. Although found in lower quantities, ARG-containing phage particles were more prevalent among the Antarctic than the Mediterranean seawater samples and Antarctic bacterial communities were confirmed as their source. In the Mediterranean area, ARG-containing phages from anthropogenic fecal pollution might allow ARG transmission through the food chain. ARGs were detected in phage particles isolated from fish (Mediterranean, Atlantic, farmed, and frozen), the most abundant being β-lactamases. Some of these particles were infectious in cultures of the fecal bacteria *Escherichia coli*. By serving as ARG reservoirs in marine environments, including those with low human activity, such as the Antarctic, phages could contribute to ARG transmission between bacterial communities.

## 1. Introduction

Antibiotic resistance is one of the biggest threats to public health this century. The growing environmental presence of antibiotics and their derivative metabolites is mainly due to anthropogenic activities, such as medicine, agriculture, and industry. Antibiotics have been overused both in clinical settings and the livestock industry, including for prophylaxis, although their use as growth supplements has been prohibited in Europe since 2006 (EC Regulation No. 1831/2003) [1,2,3]. Epidemiological studies have verified that antibiotic consumption is directly connected with the emergence and dissemination of resistances [2].

Depending on their transport and persistence in soil or water, antibiotics can spread to surface water and groundwater, exerting a selective pressure on aquatic bacteria and the microbiota of aquatic animals, and leading to the emergence of resistant bacteria and the dissemination of antibiotic resistance genes (ARGs) [4,5].

A high demand for fish products has led to an increase in aquaculture in recent years [6]. However, these intensive fish production systems, with high concentrations of animals kept in limited areas of water, entail an increased risk of infectious diseases and consequently depend on the prophylactic and metaphylactic use of antimicrobials [6]. In addition to the antibiotic contamination of water bodies, which exerts a selective pressure on aquatic microbial communities, their use in the aquaculture industry causes the accumulation of antibiotic residues in fish and shellfish tissues, which in turn selects for resistances in the fish microbiota [6]. Although the presence of antibiotics, and consequently ARGs, in aquatic environments subjected to a high anthropogenic impact is well established [7], there is less information available about ARGs in areas with few human activities, such as polar aquatic environments, where ARGs have been detected [8,9].

Microorganisms have been producing natural antibiotics for billions of years to gain a selective advantage over competitors [2], and they simultaneously evolved mechanisms of antibiotic resistance [2]. As well as intrinsic resistance to antibiotics, innate to the species, bacteria may acquire resistance by mutation or by horizontal gene transfer (HGT) [3]. The most studied HGT mechanisms are conjugation and transformation, although in recent years, transduction, or gene transfer mediated by bacteriophages or phages, has attracted increasing attention as a means of ARG transmission in the environment [10,11].

Bacteriophages are the most abundant entities in the world (10^31^ phage particles have been estimated) and can be isolated from any habitat where bacteria exist [12]. The high persistence of phages in the environment enhances the efficiency of ARG dissemination in phage particles, which could, in turn, increase the chances of ARG transduction [13]. Phages can mobilize and spread ARGs by transduction, whether generalized, specialized, or lateral [14,15,16]. Phage particles carrying ARGs have been reported in different biomes, including sludge, soil, wastewater [10,11,15,17,18], marine viromes [19], and human and animal biomes [20,21,22]. Recent studies have reported the presence of ARG-carrying phage particles in vegetables and meat products classed as suitable for consumption, confirming that they can end up in food, and suggesting that their presence in feces is due to the fecal-oral route [23,24].

There is, however, no information about the occurrence of phage-associated ARGs in areas with very low human activity. The aim of this study was to assess ARG-carrying phage particles in two different environments: (i) Mediterranean coastal water, subjected to a high anthropogenic impact and (ii) Antarctic seawater, where the anthropogenic impact is low. Considering the negligible human influence in the Antarctic area, we also examined if phages induced from the bacterial fraction of marine communities could be confirmed as the source of the ARG-containing phage particles. Additionally, the impact of ARG-containing phage particles in Mediterranean seawater on the food chain was studied by analyzing their presence in marine fish, shellfish, and farmed fish.

## 2. Materials and Methods

### 2.1. Samples

**Figure 1:**
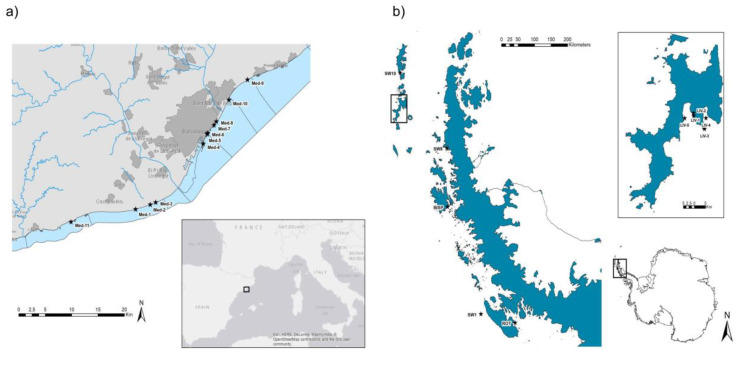
Seawater sampling sites. (**a**) Barcelona coast (Mediterranean Sea). (**b**) Livingston Island (Antarctica). Stars represent the sampling sites in each area.

Twenty seawater samples were collected from eleven different sampling sites (Med-1 to Med-11) off the Barcelona coast in February–October 2019 (Figure 1a). Ten seawater samples were collected at different sites off Livingston Island, the South Shetland Islands (LIV-1,2,3,4,5) near the Antarctic Spanish Base, and the Antarctic Peninsula (SW1, SW8, SW10, BISP and ROT) while on board the research vessel BIO Hespérides during the BLUEBIO-2 Antarctic Campaign (January–March 2019) within the Bluebio project (CTM2016-78901/ANT; Figure 1b). Antarctica seawater samples were not close to human bases or animal communities. Additionally, 50 samples of shellfish (*n* = 10 samples), Mediterranean fish (*n* = 10), Atlantic fish (*n* = 10), frozen fish (*n* = 10), and farmed fish (*n* = 10) were obtained in local supermarkets during 2018–2019. All the samples were collected in sterile containers, kept at 4 °C and analyzed within 24 h.

### 2.2. Phage Isolation from Seawater Samples

Five liters (L) of Antarctic seawater samples were filtered by 5 μm Swinnex^®^ filter holders (Millipore, Merck, Darmstadt, Germany), followed by filtration through 3 μm mixed cellulose ester membranes (MCE) (Merck), and filtration through 0.2 μm Isopore™ polycarbonate membranes. Phage particles from the final filtrate were recovered by chemical flocculation [25]. An iron chloride solution (10 g/L) was used to flocculate the viral particles in the sample, which were then recovered by filtration through 1.2 µm Isopore™ membranes. Viral particles retained in the filters were resuspended using a 0.1M EDTA-0.2M MgCl_2_-0.2M ascorbic acid buffer to a final volume of 5 mL.

For Mediterranean seawater samples, 100 mL was filtered through 0.22 μm low protein binding polyethersulfone (PES) membranes. Viral particles in filtered samples were then concentrated with PEG8000 following previously reported protocols [18,26].

Lastly, the suspensions of phages of both origins were dialyzed and concentrated with Amicon^®^ Ultra Centrifugal Filters to volumes of 0.5 mL. The concentrated phage suspensions of both origins were used for the phage DNA extraction.

### 2.3. Phage Isolation from Induced Antarctic Bacterial Communities

Antarctic seawater samples were processed within 48 h after collection for phage induction from the bacterial communities. One liter of each sample was filtered by 0.2 μm Isopore™ polycarbonate membranes (Merck) to retain the marine bacterial communities. In order to enrich the bacterial communities, filters were incubated in 7 mL of Difco™ Marine Broth at 18 °C for 48–72 h without shaking. Phages were induced from the possible lysogens present in these bacterial communities by treatment with mitomycin C (0.5 µg/mL) and incubation overnight at 20 °C in the dark without shaking [27]. After induction, phages were purified by filtration through 0.22 μm low protein binding PES membranes and concentrated with Amicon^®^ Ultra 15 mL centrifugal filters as described above. The suspensions were stored at −20 °C until processed for phage DNA extraction.

### 2.4. Phage DNA Extraction

As in previous studies [23,24], filtered and concentrated phage suspensions were treated with chloroform (1:10 (*v*/*v*)) to rule out the possible presence of vesicles containing DNA, vigorously vortexed for 5 min and centrifuged at 16,000× *g* for 5 min. The aqueous phase was incubated with DNase I (100 units/mL) to eliminate any free DNA in the samples outside the phage particles. DNase was inactivated by heating for 5 min at 75 °C.

These suspensions were then used to extract DNA from the phage capsids. Before breaking the capsids, aliquots were taken to confirm DNA removal by the DNase treatment and complete inactivation of DNase by heat treatment.

Viral capsids were broken using proteinase K (0.5 µg/mL) in 250 μL of proteinase K buffer and then incubated for 1 h at 55 °C [26]. The encapsidated DNA was extracted by phenol-chloroform (1:1) (*v*/*v*) and chloroform treatment. DNA was precipitated using 100% ethanol and 3 M sodium acetate and resuspended in 50 µL of ultrapure sterile water. DNA was quantified using a Nanodrop ND-1000 spectrophotometer.

### 2.5. Amplification of ARGs in the DNA Extracted from Phage Particles

Quantitative real-time PCR (qPCR) with TaqMan hydrolysis probes was performed using the StepOne Real Time PCR System in a 20 μL reaction mixture with the TaqMan^®^ Environmental Master Mix 2.0 (Applied Biosystems, Foster City, CA, USA). The reaction contained 9 μL of the sample DNA or standards with known DNA concentration. The results were analyzed with the Applied Biosystems StepOne™ Instrument program.

A total of 11 ARGs with a range of mechanisms and clinical relevance were evaluated. Five genes conferred resistance to β-lactam antibiotics (*bla*_TEM_, *bla*_CTX-M-1 group_, *bla*_CTX-M-9 group_, *bla*_OXA-48_ and *bla*_VIM_), two quinolone resistance genes (*qnrA* and *qnrS*), *mecA*, a gene commonly found in *Staphylococcus* that confers resistance to methicillin [15], *sul1*, which confers resistance to sulfonamides and is frequently found in environmental and clinical bacterial populations [28], *tetW*, which confers resistance to tetracycline and is commonly found and used in aquaculture environments [29], and *armA*, which encodes aminoglycoside resistance and is widely distributed in *Enterobacteriaceae* [30]. Mediterranean and Antarctic seawater samples were tested for the presence of five ARGs only (*bla*_TEM_, *bla*_CTX-M-1 group_, *bla*_CTX-M-9 group_, *tetW* and *sul1*), and the fish samples were analyzed for the eleven ARGs.

For quantification, serial dilutions of gBlocks™ Gene Fragments (Integrated DNA Technologies, Coralville, IA, USA) of known concentration were used to generate the standard curves. All samples were run in triplicate (including the standards and negative controls). The number of gene copies (GC) was defined as the mean of the triplicate data obtained. To quantify the ARGs, we considered the results obtained within the threshold cycle and within the limit of quantification. This was determined by the last valid threshold cycle for each ARG assay (GC/μL are shown in Table S1) in the standard curve that is consistent in the diverse replicates.

To confirm that only the ARGs from the phage particles were evaluated, several controls were performed as previously described [24]. Briefly, an aliquot of each sample after DNase treatment and before desencapsidation was included in the qPCR in parallel with the DNA sample to confirm the absence of each ARG. Additionally, the controls were used to confirm the absence of bacterial 16S rDNA, which was verified by qPCR using Power SYBR Green PCR Master Mix (Thermo Fisher Scientific, Waltham, MA, USA) and primers 338F/518R (Table S1).

### 2.6. Bacterial and Viral Indicators

Microbial indicators were evaluated in fish samples and Mediterranean seawater. To isolate bacteria and somatic phages from fish and shellfish samples, 20 g of each sample was homogenized for 2 min in 60 mL of phage buffer with the Stomacher homogenizer (IUL Instruments GmbH, Königswinter, Germany). Stomacher bags with filters (Afora, Barcelona, Spain) were used to enhance the separation of solid waste from the liquid fraction containing the microorganisms. For Mediterranean seawater, 100 mL of each sample was filtered through 0.45 µm mixed cellulose ester (MCE) EZ-Pak^®^ filters (Merck), which were used to analyze the bacterial indicators. Somatic coliphages were analyzed as indicated below.

Total aerobic microorganisms resistant to ampicillin (AmpR) were evaluated on Tryptone Soy Agar (TSA) with Amp (100 μg/mL) at 37 °C. Total *Escherichia coli* and *E. coli* resistant to Amp were determined on Chromocult^®^ Coliform Agar or Chromocult^®^ Coliform Agar with Amp (100 μg/mL), respectively. Incubation was first performed for 2 h at 37 °C to accommodate potentially damaged microorganisms and then overnight at 44 °C. Presumed *E. coli* colonies were confirmed in MacConkey agar. Total *Enterococcus* were enumerated by membrane filtration, according to the ISO standard method [31]. Filters were incubated for 48 h at 37 °C in Difco™ *Enterococcus* agar (BD). Suspected *Enterococcus* colonies were confirmed by growing them in bile esculin agar for 3 h at 44 °C.

Somatic coliphages were evaluated as fecal viral indicators in the samples [32]. The homogenates of fish and shellfish samples (10 mL each) were centrifuged for 15 min at 4000× *g* and the supernatant was filtered through 0.22 μm low protein binding PES membranes (Merck). Seawater samples (100 mL) were filtered through 0.22 μm low protein binding PES membranes (Merck) and concentrated with Amicon^®^ Ultra 15 mL centrifugal filters (Merck). The filtrates were analyzed in duplicate for the presence of somatic coliphages according to the ISO standard method [33], which uses *E. coli* strain WG5 (ATCC 700078) as the bacterial host, and incubated at 37 °C for 18 h.

Each sample was analyzed for all the indicators in duplicate.

### 2.7. Propagation Cultures for Phages in Fish and Shellfish Samples

The ability of phage particles in the fish and shellfish samples to infect and propagate in enrichment cultures of the *E. coli* WG5 host strain was evaluated. This strain, used for the somatic coliphage count, was selected for its sensitivity to phage infection [23] and because it does not contain any of the ARGs targeted in this study or any prophage [34].

The propagation cultures were prepared with 1 mL of each phage suspension after the DNAse treatment and 1 mL of *E. coli* WG5 at the exponential phase (OD_600_ 0.3) in 8 mL of Luria-Bertrani broth (LB) and incubated overnight at 37 °C with shaking. After incubation, phages were purified by filtration through 0.22 μm low protein binding PES membranes and treated with chloroform and DNase, as indicated above.

The infectivity of the phage particles carrying ARGs was evaluated using qPCR by comparing ARG abundance in packaged DNA before (direct quantification from the sample) and after phage propagation in the enrichment cultures. If the GC number of the ARGs in the phage particles increased after propagation, it was considered that the phage particles in this sample were able to infect and propagate in the host strain. Otherwise, if ARG levels did not vary or decreased, the particles in this sample were considered incapable of propagating.

## 3. Results

### 3.1. Comparison of ARGs in the Phage DNA Fraction from Mediterranean and Antarctic Seawater Samples

**Figure 2:**
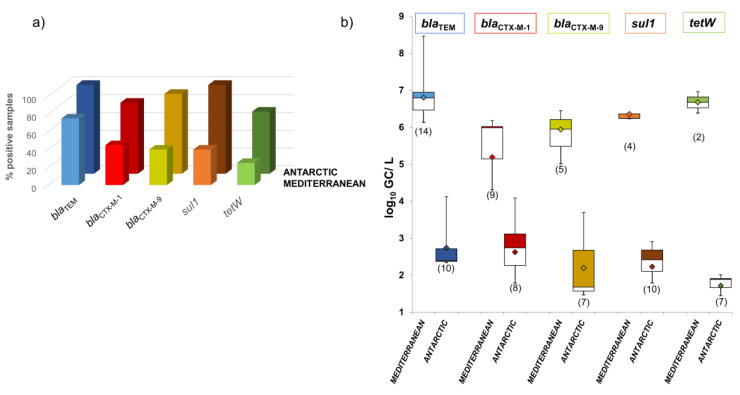
Antibiotic resistance genes (ARGs) in phage particles in Mediterranean and Antarctic seawater samples. (**a**) Percentage of positive samples for each ARG. (**b**) Abundance of each ARG in seawater (log_10_GC/L). Data are represented in boxplots; the diamond represents the average value of the positive samples, the upper squares include samples whose values are within the 75th percentile, and those in the lower white boxes are within the 25th percentile.

ARGs were detected in the phage DNA fraction isolated from Mediterranean and Antarctic seawater (Figure 2). For the five ARGs studied in both types of seawater, 25–60% fewer positive samples were obtained from the Mediterranean than the Antarctic (Figure 2a). *bla*_TEM_ was the most prevalent ARG in both sites, found in 100% of Antarctic and 75% of Mediterranean samples (Figure 2a). *sul1* was also found in 100% of Antarctic samples, but its prevalence in Mediterranean waters was much lower (40%). The second most prevalent ARG in the Mediterranean was *bla*_CTX-M-1_ (45%) but much less than in the Antarctic (80%) (Figure 2a).

In contrast with the prevalence data, the abundance of ARGs (GC/L) in the Antarctic samples was 3–5 log_10_ units lower than in the Mediterranean samples (Figure 2b). *bla*_TEM_ and *tetW* were the most abundant ARGs found in the Mediterranean, with average values close to 7 log_10_ GC/L, while *bla*_TEM_ and *bla*_CTX-M-1_ were the most abundant in the Antarctic, with average values close to 3 log_10_ GC/L.

### 3.2. ARGs in Phage Particles Induced from Antarctic Bacterial Communities

**Figure 3:**
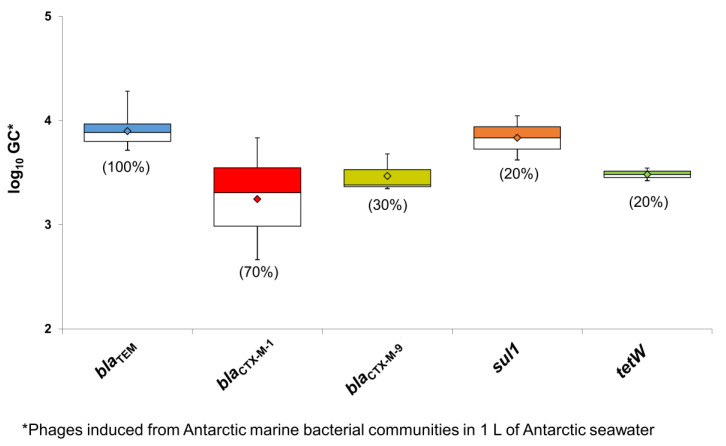
Abundance of ARGs in the phage DNA isolated from phage particles induced from Antarctic bacterial communities present in one liter of Antarctic seawater (log_10_ GC/L). Data are represented in boxplots; the diamond represents the average value of the positive samples, the upper squares include samples whose values are within the 75th percentile, and those in the lower white boxes are within the 25th percentile.

As mentioned, one of the aims of the study was to explore the origin of the ARG-containing phage particles found in the Antarctic area. We hypothesized that they could be produced by the Antarctic marine bacterial communities as part of an HGT mechanism. To test this hypothesis, the bacterial fraction of the samples was separated and treated with an inducing agent to force the production of phage particles, including those carrying ARGs. The results showed that particles carrying *bla*_TEM_ were produced in 100% of the lysates, and particles carrying *bla*_CTX-M-1 group_ were produced in 75% (Figure 3). Other ARG-containing particles were produced in a lower percentage but were still detectable.

The Antarctic ARG counts were calculated from phage particles induced from marine bacteria isolated from 1 L of seawater. Nevertheless, this abundance should be treated with caution, as the cultivation of the bacterial communities using marine broth selected the bacteria able to grow in such conditions. In addition, different phage particles may be induced at different rates. Finally, the integrity of phage particles could have been compromised during their storage from the end of the sampling campaign until phage DNA extraction in the laboratory. Despite these considerations, all ARGs in the induced phages were detected with a similar abundance. These results point to the marine bacterial communities as a plausible source of the ARG-carrying phage particles found in the Antarctic seawater.

### 3.3. Consequences of Anthropogenic Pollution in Mediterranean Environments

The higher abundance of ARG-carrying phage particles in the Mediterranean could be attributed to the anthropogenic impact as well as production by the bacterial communities. These particles could be taken up by aquatic fauna and mediate the transfer of ARGs through the food chain. To evaluate the consequences of the ARG-carrying phage particles in Mediterranean coastal waters, a set of samples of marine fish, shellfish, and farmed fish from the area were analyzed.

The microbial indicators confirmed the influence of anthropogenic pollution in seawater and in fish samples. Aerobic bacteria resistant to ampicillin were detected in all the matrices analyzed (Table 1). Predictably, the highest positive values of *E. coli* were obtained in Mediterranean seawater samples, which contained ampicillin-resistant *E. coli*. The similarity of the average values of *E. coli* and ampicillin-resistant *E. coli* in the Mediterranean samples suggests that most of the *E. coli* isolates were ampicillin-resistant. Other fecal indicators, *Enterococcus* spp., were also present in all the samples studied (Table 1), their abundance in Mediterranean seawater and fish being similar to *E. coli* counts. Somatic coliphages, used as viral indicators of fecal pollution [35], were detected in all samples except in Mediterranean fish.

### 3.4. Prevalence of ARGs in Phage DNA Fraction Isolated from Shellfish and Fish Samples

ARGs in DNA extracted from phage particles isolated from shellfish and fish were quantified by qPCR. Samples were considered positive when the amplification values of the target gene were within the limit of quantification, previously defined with the standard curves. Those samples outside the limit of quantification but within the limit of detection and those that did not show any increase in fluorescence in the qPCR cycles (undetermined samples) were excluded from the ARG prevalence study.

**Figure 4:**
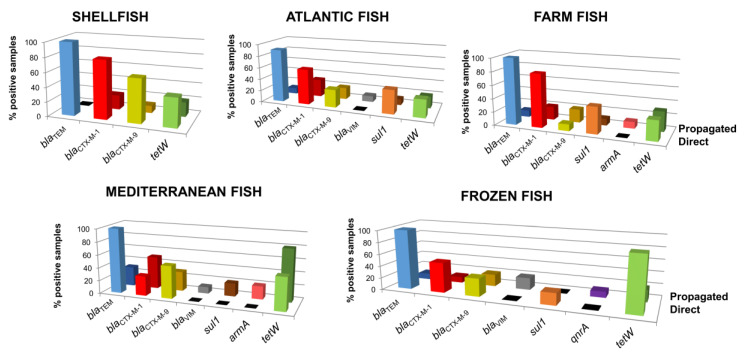
Percentage of positive samples for each ARG in each fish matrix and shellfish analyzed directly from the samples (light color bars) and after propagation in the *E. coli* enrichment culture (dark color bars).

All samples were analyzed for ARGs, either directly or after propagation in *E. coli* cultures, with 10–100% of samples testing positive (Figure 4). An increase in ARG count in the DNA after the enrichment step revealed that the phage particles were able to infect a fecal strain and propagate, and consequently a fraction of the particles in these samples were infectious. On the contrary, in samples without an increase in GC numbers or no detection of ARGs (not shown in Figure 4), the isolated phage particles were likely non-infectious or the propagation strain was not a suitable host.

Although the different matrices gave heterogeneous results, β-lactamase genes and *tetW* were the most detected ARGs in all the samples. Among the β-lactamase genes, *bla*_TEM_ was the most prevalent, found in 90–100% of samples, followed by *bla*_CTX-M-1_ and *bla*_CTX-M-9_, which were identified in 10–80% of the samples. *bla*_VIM_ was only detected in Atlantic, Mediterranean and frozen fish after propagation. Using direct analysis, *tetW* was detected in 3–90% of the samples, with the highest prevalence in frozen fish. *sul1* was determined in 20–40% of Atlantic, farmed and frozen fish samples by direct analysis and in Mediterranean fish after propagation.

The matrices with the widest range of ARGs were Mediterranean fish and frozen fish (seven in each). After the enrichment step, five ARGs were detected in an increased number of samples of Mediterranean fish, three of them (*bla*_VIM_, *sul1* and *armA*) only after propagation (Figure 4). The only matrix in which *qnrA* was detected was frozen fish, with one positive sample after propagation, which also tested positive for *bla*_TEM_, *bla*_CTX-M-1_, *bla*_CTX-M-9_, *bla*_VIM_ and *tetW*. *armA* was only detected after propagation in two Mediterranean fish samples and in one farmed fish sample. Finally, *mecA*, *qnrS*, and *bla*_OXA-48_ were not detected in any samples.

### 3.5. Abundance of ARGs in the Phage DNA Fraction Isolated from Shellfish and Fish Samples

**Figure 5:**
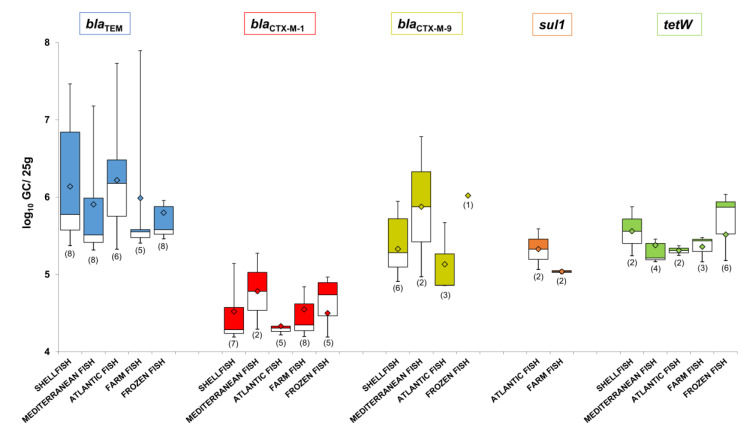
Abundance of ARGs in the DNA isolated from the phage particles of each fish matrix and shellfish (log_10_ GC/25 g) grouped by ARG. Data is represented in boxplots; the diamond represents the average value of the positive samples, the upper squares include samples whose values are within the 75th percentile, and those in the lower white boxes are within the 25th percentile.

ARG abundance in shellfish and fish was evaluated only by direct analysis of the samples (Figure 5). The abundance of ARGs in phage particles obtained after propagation studies with an enrichment step cannot be considered as an absolute value and was therefore not included in the abundance study.

The most prevalent gene, *bla*_TEM_, was also the most abundant, with average values higher than 6 log_10_ GC/25 g of sample (Figure 5) and the highest value (7.9 log_10_ GC/25 g) observed in a farmed fish sample. In contrast, despite being one of the most prevalent ARGs, *bla*_CTX-M-1,_ was the least abundant, detected at less than 5 log_10_ GC/25 g in most of the samples tested. The second most abundant ARG was *bla*_CTX-M-9_, particularly in Mediterranean and frozen fish, with average values close to 6 log_10_ GC/25 g. A similar level of abundance was found for the prevalent gene *tetW*, with the highest value of 6 log_10_ GC/25 g obtained in frozen fish samples, 90% of which were positive for this ARG. The *sul1* gene was detected in both Atlantic and farmed fish at average values higher than 5 log_10_ GC/25 g.

Comparing the abundance of all ARGs in the phage fraction of each type of fish reveals higher values in shellfish, Mediterranean, and Atlantic fish than in farmed and frozen fish, with some exceptions (Figure S1).

## 4. Discussion

Antibiotic resistance is as ancient as some of the earliest bacterial communities, but little is known about the presence and persistence of antibiotics in the environment before they were harnessed for the benefit of humanity [36]. Seawater from the Antarctic, an area with a presumably limited anthropogenic influence, was expected to have a low prevalence of ARGs, but surprisingly, it was found to be higher than in Mediterranean samples. An explanation for these results could be the difference in sample volumes analyzed. Following reported protocols for environmental samples [18,25], larger volumes of seawater were collected and processed from the Antarctic to compensate for the expected limited ARG presence. We are aware that the volume affects the limit of detection, and the use of higher volumes of Mediterranean seawater could have led to more positive samples. Nevertheless, when a Mediterranean sample was positive, the abundance of quantified ARGs was much greater than in the Antarctic samples, being around 4 log units more with 50 times less volume, suggesting that ARGs in the Mediterranean samples are unlikely to have been underestimated. Independently of the number of positive samples, the presence of the same ARGs in both ecosystems is striking. In natural ecosystems, the production of antibiotics, innate in many bacterial communities, is a protective mechanism against competitors. In 2011, a study demonstrated the presence of ARGs in bacterial populations isolated from permafrost sediments, supporting that ARGs pre-date the human use of antibiotics and that antibiotic resistance occurs naturally in nature [37].

The most prevalent ARGs in Antarctic seawater were β-lactamase genes and *sul1*, in agreement with previous reports of β-lactamase genes (especially from CTX-M groups) in remote polar areas [38]. There was a notable presence of *sul1*, which expresses resistance to synthetic or semi-synthetic antibiotics such as sulphonamides. A recent study detected resistance against these antibiotics in bacteria recovered from freshwater samples collected close to Antarctic research stations [39], which was attributed to poor waste water treatment. Nevertheless, *sul1* is not necessarily an indicator of human activity, as it is usually present in environmental bacteria from soils and waters, even in the Antarctic [40]. Furthermore, *sul1* is associated with an integron, which could explain its wide dissemination even in the absence of selective pressure by antibiotics [41].

Despite the samples analyzed from Antarctica were not close to human bases, the growing human activity in the Antarctic area, including scientific expeditions, military stations, and tourism [42], could be responsible for disseminating human-origin antibiotic resistance [38]. Another contributing factor may be migrating wildlife, which by connecting anthropogenic and remote areas could act as biological vectors spreading antibiotic resistant bacteria and ARGs to the Antarctic [36].

The greater abundance of ARGs in the Mediterranean samples can be explained by a high antibiotic selective pressure derived from human activity in the Mediterranean coast and the impact of human fecal pollution. These results are in line with the relative abundance of ARGs recently reported by Calero-Cáceres et al., [19] in the viromes of samples from diverse marine habitats. Specifically, the diversity and prevalence of phage ARGs in Indian Ocean samples, like the Mediterranean samples studied here, were relatively low, yet the abundance of certain genes was high, particularly those conferring resistance to β-lactams (especially *bla_TEM_*) and tetracyclines. Although a clear association has not yet been established, these findings shed light on the selective pressure generated by humans in natural environments, where bacteria can take advantage of phages to spread their genetic content in difficult conditions.

The ARGs detected in the phage DNA fraction isolated from lysogens of Antarctic bacteria allow us to suggest that these bacterial communities are the source of the ARGs in the phage particles. The particles would be formed after the packaging of bacterial DNA (including ARGs) by prophages inserted in the bacterial chromosome. Several studies reveal that temperate phages are widely distributed in Antarctic bacterial communities and prioritize lysogeny when bacterial concentration is low [43].

Our results therefore confirm the existence of a diversity of ARGs in an area as pristine as the Antarctic, and that bacteriophages in marine environments represent a potential vehicle for the horizontal transmission of ARGs between bacterial communities. The role of phages as HGT elements in marine environments has already been demonstrated, as they can disseminate key genes involved in oxygenic photosynthesis in cyanobacteria, which allows various bacterial communities to adapt to surface waters [44]. The demonstrated ability of phages to interact with their hosts has evolutionary and ecological implications.

On the other hand, the high abundance of phage-associated ARGs found in Mediterranean seawater may have important consequences for fish and shellfish, as well as consumers of fishery products. According to our results, fish microbiota showed a high percentage of resistance to β-lactam antibiotics, which represents both a direct health risk to consumers as well as a general health hazard if this resistance is transferred to human microbiota. These results are in accordance with a recent study in which ARGs were detected in wild fish, the most prevalent being *bla*_TEM_ [45].

The most commonly used antibiotics in aquaculture are fluoroquinolones, sulfonamides, and tetracyclines [29]. The prevalence of *tetW* in our samples may reflect the overuse of tetracyclines in aquaculture and their spread in diverse environments [46]. Although the sulfonamide resistance gene *sul1* has been reported as one of the most prevalent ARGs in phage DNA of environmental and food samples [7,23,24], in the current study it was detected only in a few samples of fish, being more prevalent in farmed fish, in accordance with the use of sulfonamides in aquaculture [47].

European food safety regulations establish that foodstuffs should not contain microorganisms in quantities representing a risk for human health [48], The samples analyzed in the current study were all suitable for consumption according to the microbiological parameters evaluated. In recreational coastal water, the European normative establishes that 500 CFU/100 mL of *E. coli* and 185 CFU/100 mL of intestinal enterococci in a sample indicate sufficient quality for consumption [49]. However, the detection of phages, including those capable of transferring ARGs to the natural microbiota and pathogens, is not contemplated in food safety practices. There is no legislation in place to control the spread of antibiotic resistance, ARGs, or the genetic elements that mobilize them.

## 5. Conclusions

This study confirms the presence of ARG-containing phage particles in aquatic environments subjected to a low or high anthropogenic influence, demonstrating that seawater is a reservoir of ARGs and that phages could play an important role in their transmission. In addition, human activity has a clear impact on the evolution of antibiotic resistance, the strongly anthropogenic Mediterranean area showing a higher abundance of ARGs than the relatively pristine Antarctic seawater. This high abundance of ARG-containing phage particles may have an important impact on fish and shellfish microbiota, contributing to the dissemination of those ARGs into microbial communities relevant to human health.

## Figures and Tables

**Table 1:** Bacterial (CFU/25 g) and viral (PFU/25 g) indicators in Mediterranean seawater, fish and shellfish samples. -; only one positive sample, no SD.

Microorganism		Mediterranean Seawater *	Fish				
			Shellfish	Mediterranean	Atlantic	Farm	Frozen
*n*		20	10	10	10	10	10
Total aerobic bacteria AmpR	% positive samples	100	90	80	70	100	40
	Average	2.4 × 10^3^	4.6 × 10^7^	5.1 × 10^4^	5.7 × 10^6^	3.8 × 10^6^	1.0 × 10^8^
	SD	5.6 × 10^3^	4.9 × 10^7^	5.9 × 10^4^	9.4 × 10^6^	8.3 × 10^6^	1.2 × 10^8^
*E. coli*	% positive samples	85	0	10	10	0	0
	Average	6.9 × 10^2^	0	3.7 × 10^1^	7.5 × 10^1^	0	0
	SD	2.1 × 10^3^	0	−	−	0	0
*E. coli* AmpR	% positive samples	60	0	0	0	0	0
	Average	6.8 × 10^2^	0	0	0	0	0
	SD	1.9 × 10^3^	0	0	0	0	0
*Enterococcus* spp.	% positive samples	90	30	10	40	20	10
	Average	1.0 × 10^2^	1.4 × 10^2^	7.5 × 10^1^	7.3 × 10^2^	1.9 × 10^2^	1.1 × 10^2^
	SD	1.5 × 10^2^	1.2 × 10^2^	−	1.2 × 10^3^	5.3 × 10^1^	−
Somatic coliphages	% positive samples	40	70	0	30	10	20
	Average	1.0 × 10^3^	1.1 × 10^3^	0	9.2 × 10^1^	7.5 × 10^1^	6.6 × 10^2^
	SD	1.3 × 10^3^	9.9 × 10^2^	0	5.2 × 10^1^	-	8.2 × 10^2^

* CFU or PFU/L.

## Supplementary Materials

The following are available online at https://www.mdpi.com/2076-2607/8/9/1293/s1, Figure S1: Abundance of ARGs in the DNA isolated from phage particles in fish and shellfish grouped by fish matrix. Table S1: Oligonucleotides used in this study [50,51,52,53,54].
