# Supplementary material for: Antibiotic Resistance Genes in Phage Particles from Antarctic and Mediterranean Seawater Ecosystems
Source: Microorganisms. 2020 Aug 24;8(9):1293. doi: 10.3390/microorganisms8091293 (PMC7565065; doi:10.3390/microorganisms8091293)
Supplement: Supplementary file 1 [file microorganisms-08-01293-s001.pdf]

Supplementary File

Antibiotic Resistance Genes in Phage Particles from Antarctic and Mediterranean Seawater Ecosystems

Pedro Blanco-Picazo [1], Gabriel Roscales [1], Daniel Toribio-Avedillo [1], Clara Gómez-Gómez [1], Conxita Avila [2], Elisenda Ballesté [1], Maite Muniesa [1,*] and Lorena Rodríguez-Rubio [1,*]

[1] Department of Genetics, Microbiology and Statistics, University of Barcelona, Diagonal 643, Annex, Floor 0, 08028 Barcelona, Spain; p.blanco@ub.edu (P.B.-P.), gascheb@gmail.com (G.R.), d.toribio.avedillo@ub.edu (D.T.-A.), claragg1995@gmail.com (C.G.-G.), eballeste@ub.edu (E.B.)

[2] Department of Evolutionary Biology, Ecology and Environmental Sciences (BEECA), Faculty of Biology, and Biodiversity Research Institute (IrBIO), University of Barcelona, Diagonal 643, 08028 Barcelona, Spain; conxita.avila@ub.edu

* Correspondence: mmuniesa@ub.edu (M.M.); lorenarodriguez@ub.edu (L.R.-R.)

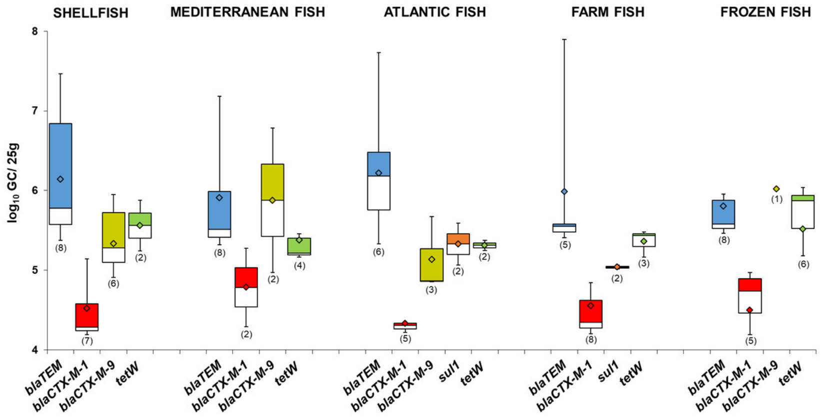

Figure S1. Abundance of ARGs in the DNA isolated from phage particles in fish and shellfish grouped by fish matrix. Data is represented in boxplots (log_{10}GC/25 g). Percentage represents the number of positive samples detected. The diamond represents the average value of the positive samples, the upper squares include samples whose values are within the 75th percentile, and those in the lower white boxes are within the 25th percentile.

Table S1. Oligonucleotides used in this study.

Target Gene	Oligonucleotide	Sequence	Amplimer (bp)	LOQ (GC/µl)	Reference
*bla*TEM	UP	CACTATTCTCAGAATGACTTGGT	85	2.4	[50]
	LP	TGCATAATTCTCTTACTGTCATG			
	TaqMan TEM	6FAM-CCAGTCACAGAAAAGCATCTTACGG-MGBNFQ			
*bla*CTX-M-1-group	UP	ACCAACGATATCGCGGTGAT	101	0.2	[15]
	LP	ACATCGCGACGGCTTTCT			
	TaqMan CTX-M-1	6FAM–TCGTGCGCCGCTG-MGBNFQ			
*bla*CTX-M-9-group	UP	ACCAATGATATTGCGGTGAT	85	1.0	[22]

	LP	CTGCGTTCTGTTGCGGCT			
	TaqMan CTX-M-9	6FAM – TCGTGCGCCGCTG- MGBNFQ			
*bla*OXA-48	UP	CGGTAGCAAAGGAATGGCAA	133	18.4	[20]
	LP	TGGTTCGCCCGTTTAAGATT			
	TaqMan OXA-48	6FAM-CGTAGTTGTGCTCTGGA-MGBNFQ			
*bla*VIM	UP	AATGGTCTCATTGTCCGTGATG	61	22.5	[23]
	LP	TCGCACCCCACGCTGTA			
	TaqMan VIM	6FAM –TGATGAGTTGCTTTTGATTG-MGBNFQ			
sul1	UP	CCGTTGGCCTTCCTGTAAAG	67	1.5	[17]
	LP	TTGCCGATCGCGTGAAGT			
	TaqMan sul1	6FAM-CGAGCCTTGCGGCGG-MGBNFQ			
mecA	UP	CGCAACGTTCAATTTAATTTTGTTAA	92	11.7	[51]
	LP	TGGTCTTTCTGCATTCCTGGA			
	TaqMan mecA	6FAM-AATGACGCTATGATCCCAATCTAACTTCCACA-MGBNFQ			
qnrA	UP	AGGATTGCAGTTTCATTGAAAGC	138	0.9	[52]
	LP	TGAACTCTATGCCAAAGCAGTTG			
	TaqMan qnrA	6FAM-TATGCCGATCTGCGCGA-MGBNFQ			
qnrS	UP	CGACGTGCTAACTTGCGTGA	118	2.0	[52]
	LP	GGCATTGTTGGAAACTTGCA			
	TaqMan qnrS	6FAM-AGTTCATTGAACAGGGTGA-MGBNFQ			
armA	UP	GAAAGAGTCGCAACATTAAATGACTT	94	8.0	[53]
	LP	GATTGAAGCCACAACCAAAATCT			
	TaqMan armA	6FAM-TCAAACATGTCTCATCTATT-MGBNFQ			
tetW	UP	GACGGACACCATGTTTTTGGA	62	1.9	This study
	LP	AGGAAGTGACTGCCGCTTGA			
	TaqMan tetW	6FAM-AGCGTGGGATTACCA- MGBNFQ			
16SrDNA	338F	ACTCCTACGGGAGGCAGCAG	236		[54]
	518R	ATTACCGCGGCTGCTGG			
pGEM	pGEM7up	TGTAATACGACTCACTAT			Promega
